# Gastrointestinal Permeation Enhancers Beyond Sodium Caprate and SNAC ‐ What is Coming Next?

**DOI:** 10.1002/advs.202400843

**Published:** 2024-06-17

**Authors:** Marilena Bohley, Jean‐Christophe Leroux

**Affiliations:** ^1^ Institute of Pharmaceutical Sciences Department of Chemistry and Applied Biosciences ETH Zurich Zurich 8093 Switzerland

**Keywords:** bioavailability, intestinal permeation enhancers, oral peptide delivery, SNAC, sodium caprate

## Abstract

Oral peptide delivery is trending again. Among the possible reasons are the recent approvals of two oral peptide formulations, which represent a huge stride in the field. For the first time, gastrointestinal (GI) permeation enhancers (PEs) are leveraged to overcome the main limitation of oral peptide delivery—low permeability through the intestinal epithelium. Despite some success, the application of current PEs, such as salcaprozate sodium (SNAC), sodium caprylate (C8), and sodium caprate (C10), is generally resulting in relatively low oral bioavailabilities (BAs)—even for carefully selected therapeutics. With several hundred peptide‐based drugs presently in the pipeline, there is a huge unmet need for more effective PEs. Aiming to provide useful insights for the development of novel PEs, this review summarizes the biological hurdles to oral peptide delivery with special emphasis on the epithelial barrier. It describes the concepts and action modes of PEs and mentions possible new targets. It further states the benchmark that is set by current PEs, while critically assessing and evaluating emerging PEs regarding translatability, safety, and efficacy. Additionally, examples of novel PEs under preclinical and clinical evaluation and future directions are discussed.

## Graphical abstract

This review focuses on the main challenge of oral peptide delivery ‐ overcoming the epithelial barrier. With many peptide drugs in development, there is a huge unmet need for more efficient intestinal permeation enhancers (PEs). Biophysiological fundamentals are explained while highlighting possible new targets. Furthermore, the performance parameters of conventional PE are discussed, and how novel concepts can contribute to addressing key challenges in this field are explored.

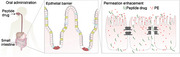

## 1 Introduction

The recent commercialization of two oral peptide formulations could be seen as a breakthrough in oral peptide delivery. In 2019, the Food and Drug Administration (FDA) approved the oral dosage form of semaglutide (Rybelsus) developed by Novo Nordisk for the treatment of type 2 diabetes^[^ ^1^ ^]^ followed by the octreotide capsule (Mycapssa), developed through Chiasma's Transient Permeation Enhancer (TPE) technology for the treatment of acromegaly in 2020.^[^ ^2^ ^]^ These two oral peptide products represent a milestone in the field because formulations allowing the successful oral delivery of macromolecular peptide drugs have been desired for decades.

Peptide‐based drugs offer tremendous therapeutic potential for a broad variety of ailments, including cancer, metabolic, and cardiovascular diseases.^[^ ^3^ ^]^ To date, there are already over 80 peptide drugs approved, >150 compounds in clinical development, and over 600 in preclinical studies. However, most peptide‐based therapeutics have one major drawback: they must be administered via injection, either intravenously (i.v.), subcutaneously (s.c.), or intramuscularly (i.m.).^[^ ^4^ ^]^ While oral administration is the most convenient, patient‐friendly, and easiest mode of drug delivery (**Table** **1**), it is most of the time characterized by low peptide BA and subtherapeutic concentrations.^[^ ^5^ ^]^

**Table 1:** Benefits of oral peptide delivery over conventional parenteral administration.

– Ease of use: it does not require any expertise, special equipment, or trained medical personal.
– Convenience and patient acceptance favor adherence and compliance, especially for treatments that are used chronically and require frequent dosing.
– Avoidance of certain side effects such as pain and discomfort, scarring, (allergic) reactions at the injection site, and cutaneous infections associated with injections.
– Reduction of healthcare expenditures by removing the need for complex auto‐injectors or healthcare professionals to deliver parental formulations.
– Reformulation into an oral formulation can expand the commercial life cycle for marketed injectable peptide‐based drugs and can generate large market sales.
– No need for sterilization (might not apply for microneedle‐based formulations), potentially offsetting higher costs associated with large quantities of peptide needed.
– Oral peptide formulations can be used for new therapeutic indications previously not considered due to the frequency of administration and/or psychological injection barrier (fear of needles).

Upon oral administration, the absorption of most peptide drugs is strongly limited by GI barriers formed by digestion, mucus, and the epithelium. Huge efforts have been made to overcome these hurdles and ultimately increase oral BAs.^[^ ^6^, ^7^ ^]^ A plethora of technologies using physical modes such as direct injection, jetting, ultrasounds, and iontophoresis have been investigated to overcome the intestinal epithelial barrier. Numerous recent reviews provide a comprehensive overview of this field.^[^ ^8^, ^9^, ^10^, ^11^, ^12^, ^13^, ^14^ ^]^ While some physical approaches revealed promising preclinical and clinical results regarding the enhancement of oral BAs of peptide therapeutics, the translatability remains, at this stage, questionable due to complexity, issues with reliability, uncertain regulatory pathways, and potential safety concerns.

To date, the most extensively investigated and most successful approach remains the use of PEs to increase the permeability of the epithelial barrier.

PEs are an inhomogeneous class of substances ranging from small molecules to biologics that transiently alter the GI epithelial barrier to facilitate permeation of macromolecules.^[^ ^15^ ^]^ Rybelsus and Mycapssa are formulated with the PEs sodium salcaprozate (SNAC) as a tablet and sodium caprylate (C8) as a lipidic capsule, respectively. They are, so far, the only marketed oral peptide‐based drugs relying on a PE‐based absorption process.^[^ ^1^, ^2^ ^]^ Even though PEs have been intensively studied and many have been shown to efficiently increase GI permeability in pre‐clinical settings, only a few have progressed to clinical trials. Of those, modest, single‐digit (mostly around 1–5%, highest 9%) increases in oral BAs of peptides with high variability have been achieved.^[^ ^15^, ^16^ ^]^ While average BA values of ca. 1% were sufficient for semaglutide and octreotide, for other peptides this might be too low, calling for the development of more potent PEs.^[^ ^17^, ^18^ ^]^ Next‐generation PEs must be compatible with a wide range of macromolecular drugs, should ideally approach oral BAs in the double‐digit range (10%), be safe with a transient mode of action, and, in a perfect scenario, deliver precise and reproducible peptide doses. If successful, these advances would completely break open the field of oral peptide delivery.^[^ ^6^, ^7^ ^]^

Here, we describe the biological barriers to oral peptide delivery with a special focus on the epithelium, summarize peptide characteristics that favor oral administration, comprehensively discuss possible modes of PE action, and highlight new targets. Finally, achievements and limitations of current PEs are outlined and approaches under preclinical or clinical investigation are critically assessed and evaluated in regard to translatability, safety, and efficacy. Overall, this review aims to provide a critical yet focused perspective on the field of intestinal PEs, commenting on translatability, outlying general controversies, highlighting recent advantages, and ultimately suggesting further directions.

## 2 Barriers to Oral Peptide Delivery

**Figure 1:**
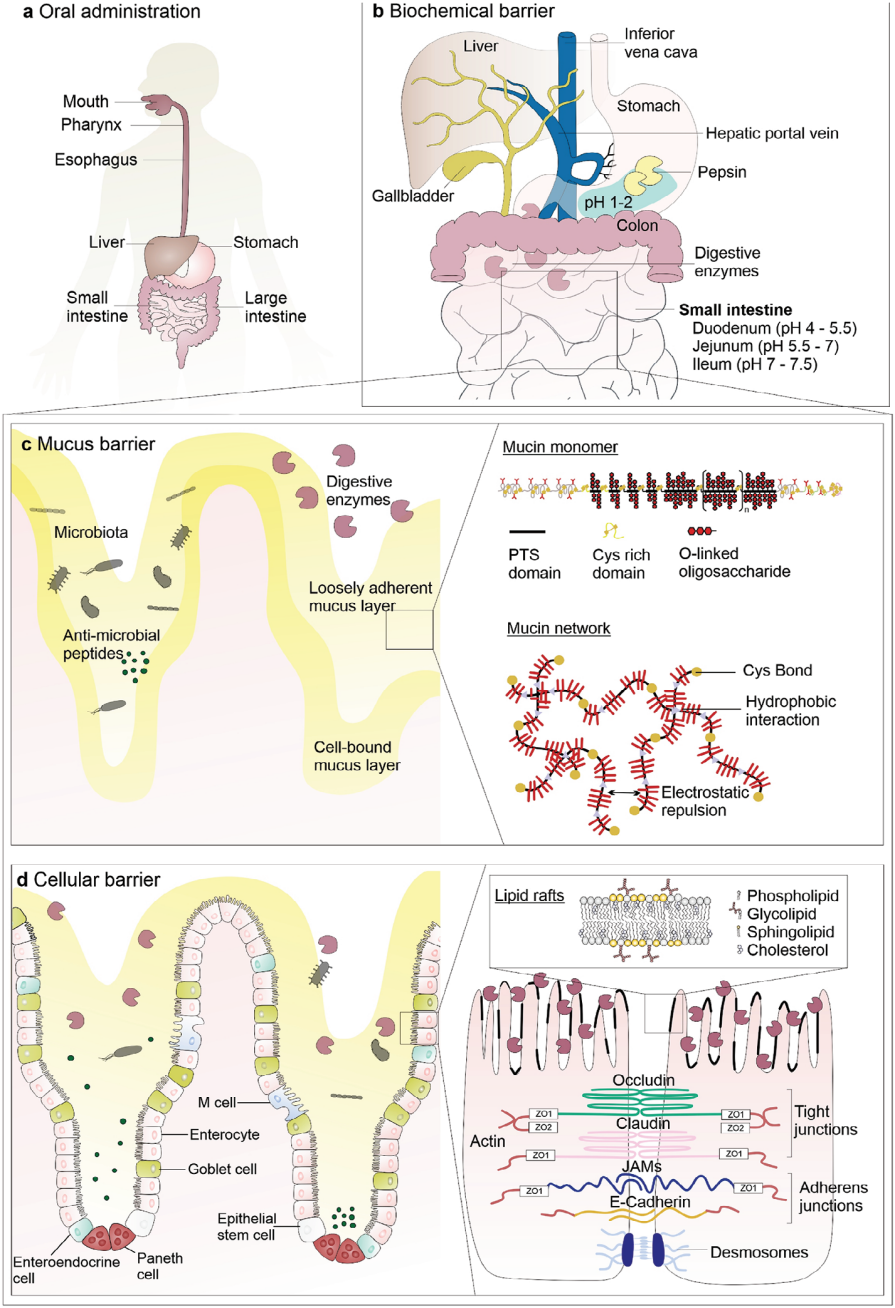
Barriers to oral peptide delivery. Various biological barriers limit the BA of orally administered peptides. a) Organs encountered by peptides after oral administration. b) Biochemical barrier: low gastric pH and digestive enzymes including pepsin in the stomach, trypsin, chymotrypsin, and carboxypeptidase in the small intestine. c) Mucus barrier: a hydrogel‐like substance formed by highly glycosylated proteins (mucins) coating the entire GI tract, creating a physical barrier. Cys: cysteine; PTS domains: tandem repeats of the amino acids proline (P), ‐threonine (T), and ‐serine (S). d) Cellular barrier: the tight epithelium strictly regulates the transport of nutrients and proteins between the gut lumen and the bloodstream. Tight junctions (TJs), adherens junctions (AJs), and desmosomes between adjacent epithelial cells physically prevent paracellular transport. Transport through epithelial cells is also hindered due to degradation by brush boarder enzymes and by limited permeation through lipid rafts.

The challenges of oral peptide delivery become evident when considering the complexity and efficiency of the GI tract's physiological functions (**Figure** **1**a). The GI tract is built to digest nutrients while simultaneously preventing the entry of foreign particulates and potential pathogens. The mucus barrier and the intestinal epithelium restrict the access of the latter.^[^ ^5^, ^19^ ^]^ Consequently, most orally administered peptides must successfully overcome the digestion, mucus, and epithelial barrier prior to their absorption.

### 2.1 Biochemical Barrier

The biochemical or digestion barrier is mainly presented by digestive enzymes and drastic changes in pH (Figure 1b). Proteases and other proteolytic enzymes readily cleave proteins at specific cleavage sites and are located throughout the whole GI tract. However, they are most prevalent in the stomach and the small intestine.^[^ ^20^ ^]^ Gastric glands in the stomach produce a highly efficient digestive cocktail (pH 1–2) containing hydrochloric acid and pepsin. Pepsin has its pH optimum at 1.6, acts as a broad endopeptidase, and is highly efficient at proteolysis.^[^ ^21^ ^]^

Upon entering the small intestine, digestion continues. While the small intestine is responsible for most absorption, it also plays a key role in the metabolism of nutrients. For peptides, the most important digestive enzymes are proteases stemming from pancreatic secretions and the brush border membrane of enterocytes. The three most abundant proteases are trypsin, α‐chymotrypsin, and carboxypeptidase.^[^ ^22^, ^23^ ^]^ For most peptide‐based drugs, inactivation of and/or protection from these enzymes and pH changes is essential to achieve effective oral delivery.^[^ ^6^ ^]^ For example, contact with the acidic stomach content can be avoided using enteric formulations, which release the drug in a controlled manner upon reaching higher pH values in the small intestine.^[^ ^24^ ^]^ The peptide structure can be optimized to enhance GI tract stability and reduce degradation by digestion.^[^ ^3^, ^4^, ^25^, ^26^, ^27^, ^28^, ^29^, ^30^, ^31^, ^32^ ^]^ Adding protease inhibitors to the final oral drug formulation can further help to prevent a certain degree of degradation by digestive enzymes.^[^ ^7^, ^28^ ^]^

### 2.2 Mucus Barrier

The physiological function of mucus is to protect epithelial surfaces by efficiently trapping and clearing pathogens and foreign particulates. This feature concurrently hinders the diffusion of peptide drugs and further facilitates clearance from the delivery site (Figure 1c). Consequently, mucus is another important barrier limiting the BA of orally administered macromolecular drugs.^[^ ^33^, ^34^ ^]^ It consists of a complex, viscoelastic, hydrogel‐like substance that is secreted by goblet cells. The main protein and gel‐forming components are mucins (MUCs), large heavily glycosylated glycoproteins. There are two major types of MUCs, which are either monomeric, cell‐bound, and located at the epithelial cell surface (MUC1, MUC3, MUC4, MUC12, and MUC13), or oligomeric and secreted (MUC2, MUC5AC and MUC6).^[^ ^35^ ^]^ MUC2, which is the predominant form in the intestine, is mainly responsible for the barrier properties of mucus. Structurally, gel‐forming MUCs exhibit a long flexible protein backbone with a high number of PTS domains.^[^ ^35^ ^]^ The hydroxyl groups of these PTS domains provide sites for O‐glycosylation. The resulting oligosaccharide side chains cause the protein to stiffen, expand the volume domain of the molecules, and induce the formation of intra‐ and inter‐mucin interactions. Flanks of the polypeptide backbone are additionally equipped with a cysteine‐rich region to facilitate disulfide bonds. Together, a 3D mesh‐like network with unique viscoelastic and space‐filling properties is generated.^[^ ^36^ ^]^ Even though the average mesh‐size has been estimated to be ca. 20–200 nm, mucus has been shown to hamper the diffusion of molecules with sizes far below. The diffusion of molecules through the mucus layer is also controlled by intermolecular covalent and non‐covalent interactions.^[^ ^37^, ^38^ ^]^ However, the extent to which peptide drug permeation through mucus is limited in vivo remains unclear.^[^ ^6^ ^]^

Besides water, lipids, and electrolytes, mucus contains antimicrobial peptides, protease inhibitors, and various other active proteins.^[^ ^33^, ^36^ ^]^ It also provides a nutrient‐rich environment for commensal bacteria, which colonize the GI tract. Even though the role of these bacteria in oral peptide delivery is poorly understood, it is well known that organisms such as *Streptococcus, Lactobacillus, Bacteroides*, and *Clostridiales* significantly contribute to the digestion and metabolism of proteins.^[^ ^39^, ^40^ ^]^

### 2.3 Cellular Barrier ‐ The Intestinal Epithelium

Upon crossing the mucus layer, peptides face the major barrier to oral drug delivery presented by the intestinal epithelium (Figure 1d), a continuous, polarized monolayer formed by goblet cells, enteroendocrine cells, Paneth cells, microfold (M) cells, and enterocytes that separates the gut lumen from the blood stream.^[^ ^41^ ^]^ Goblet cells are responsible for mucus production and secrete MUCs. Enteroendocrine cells react to external stimuli by secreting various mediators and reactive proteins such as the glucagon‐like peptide 1 (GLP‐1), gastric inhibitory polypeptides, and somatostatin. Paneth cells release antimicrobial peptides, aiming to protect nearby stem cells, which are responsible for the steady renewal of the epithelium (every 2–6 days). M cells, which make up 5–10% of the intestinal cells, play a crucial role in the immune response and defense by facilitating the transport of antigens and microorganisms from the gut lumen to the underlying immune cells. Together with these immune cells, they are part of the so‐called Peyer's patches. The latter is the immune sensors of the intestine, that can induce immune tolerance or defence mechanisms against pathogens.^[^ ^41^, ^42^, ^43^ ^]^ Enterocytes, which represent the main cell population (> 70%), are responsible for processing and moving nutrients from the luminal space into the systemic circulation. For efficient nutrient uptake either via diffusion or active transport, their apical surface is covered with microvilli building the brush border.^[^ ^44^, ^45^ ^]^ These actin‐based protrusions extend from the surface of the enterocytes into the lumen, housing various membrane‐bound enzymes, including peptidases and glycosidases, transporters, and channels. At this point, it is noteworthy to mention that luminal peptide degradation accounts for only 20% of the degradation of ingested proteins, with the brush border enzymes constituting the predominant portion.^[^ ^46^, ^47^ ^]^ The two main functions of the brush‐border are to provide a digestive and absorptive surface and to act as a protective barrier hindering the permeation of pathogens. To fulfill these tasks and withstand the constant exposure to hepatic bile salts, pancreatic proteases, and lipases, the brush‐boarder is a highly stable membrane with a unique composition. Stability and resistance are achieved by lipid rafts which are liquid‐ordered domains in the cell membrane that contain high amounts of glycolipids, cholesterol, and sphingolipids.^[^ ^46^, ^48^, ^49^ ^]^ These unique features make the epithelial cell brush boarder a prominent barrier for efficient absorption of peptides.^[^ ^50^ ^]^ Orally administered peptide drugs can enter the bloodstream via the paracellular route (through intercellular spaces between adjacent cells) or the transcellular route (transit through cells). Both ways face challenges, as the epithelium is specialized to strictly regulate the transport of molecules from their apical to their basolateral membrane.^[^ ^51^ ^]^ The paracellular passage is restricted by a complex network of molecular barriers, including TJ protein complexes, AJs, and desmosomes. TJs are pivotal for maintaining cellular polarity and regulating paracellular permeability. TJs are multiple unit structures that interact with the underlying apical actomyosin ring. Central to TJs are TJ‐proteins such as claudins and occludins.^[^ ^52^ ^]^ Claudins occupy a crucial position at the apical neck of polarized intestinal epithelial cells, serving as the cornerstone for establishing the paracellular barrier properties. So far, >25 proteins have been allocated to the claudin protein family. Their distribution is tissue‐specific. Claudin‐1, −2, −3, −4, and −7 are commonly found in the intestine and play critical roles in maintaining intestinal barrier function.^[^ ^53^, ^54^ ^]^ A common feature is that their PDZ domain (a common structural motif found in proteins allowing protein‐protein interaction) at the C‐terminus facilitates interaction with PDZ proteins of Zonula occludens (ZO) −1, −2, and −3. In contrast, occludin interacts with ZO proteins through a PI3K domain.^[^ ^55^ ^]^

ZO proteins significantly influence TJ and AJ formation pathways. The scaffolding proteins manage binding and ensure the expression of cytoskeleton and transmembrane components. Their involvement extends to gene transcription, cell proliferation, claudin polymerization management, and cadherin cell‐cell adhesion promotion. ZOs are activated via phosphorylation by protein kinase C (PKC) and tyrosine kinase.^[^ ^55^, ^56^, ^57^ ^]^ Through ZOs, TJs are associated with calcium ion (Ca^2+^)‐ and ATP‐dependent actomyosin filaments which are composed of actin and myosin. One mechanism to regulate TJs is through phosphorylation/dephosphorylation of the myosin light chain (MLC) via kinases/phosphatases, leading to the contraction/relaxation of the actomyosin ring. Elevated MLC phosphorylation increases TJ permeability. Actin filament contraction is regulated by Ca^2+^ and Rho kinase. Ca^2+^ channels are vital for allowing extracellular Ca^2+^ into the cell. Without this influx, actin filaments remain relaxed, compromising the cellular barrier structure.^[^ ^58^ ^]^ However, it has been shown that even when actin remains relaxed, the barrier function is in part maintained.^[^ ^59^ ^]^

Taken together, the highly regulated interplay between different proteins forms a seal between adjacent epithelial cells. The negatively charged protein complexes have an estimated average pore diameter of ca.1 nm (8–13 Å). Therefore, they can hamper and/or prevent paracellular transport of most peptide drugs, even very small peptides (500–1000 Da; ca. 1–2 nm).^[^ ^60^, ^61^, ^62^, ^63^ ^]^ However, they selectively allow paracellular transport, regulating trans‐TJ flux via two different mechanisms, the pore pathway, and the leak pathway. While claudins are important in regulating the pore pathway, ZO‐1 and occludin are key players in the leak pathway. Regulation of these pathways occurs by interactions between multiple classes of TJ proteins. There might also be cross‐talk between the pore and leak pathways via intracellular and extracellular signaling mechanisms such as kinase activation and cytokine release by non‐epithelial cells. Overall, the high‐capacity pore pathway allows small, uncharged solutes and specific ions to pass, while the low‐capacity leak pathway is permeable to larger macromolecules but is not ion‐selective. Yet, the specific contributions of individual proteins to TJ and AJ function remain to be fully elucidated and the resulting implication for oral peptide delivery is still not understood.^[^ ^64^ ^]^

The other route peptides can take to reach blood circulation is transcellular transport. In some cases, proteins are shuttled to the opposite membrane via transcytosis. However, if recognized as a foreign protein, they can either be subjected to the lysosomal pathway, which can lead to degradation, or be rerouted back to the mucosal surface and secreted into the small intestinal lumen.^[^ ^44^, ^60^, ^63^ ^]^ In contrast to the transcellular/transmembrane route, transcytosis is an active, receptor‐mediated process. Targeting enterocytes, requires binding at the brush border membrane, endocytosis, membrane trafficking through the endosomal compartments, and finally the release from the basolateral cell surface. Theoretically, M cells are an attractive target due to their function to sample and transport antigens, however, transport efficacy is strongly limited by the small percentage of cells. Despite these limitations, both pathways are increasingly gaining attention in the context of lymphatic transport. The idea is based on the fact that entities entering the lymphatic system are delivered directly to the systemic circulation while avoiding hepatic first‐past metabolism. Efficacy is constrained by the necessity for drug conjugates or sophisticated drug delivery systems. These systems must either facilitate integration into chylomicrons through endocellular processes inside enterocytes (for highly lipophilic compounds; high logP), followed by release into the lymph and transfer into the systemic circulation, or enable active uptake into M cells and transcytosis. In the last case, they should also bypass or evade immunocytes ‐ ideally without triggering immune responses.^[^ ^65^ ^]^ Detailed descriptions of the different active/receptor‐mediated transport mechanisms as well as advantages and challenges in harnessing these pathways using advanced drug delivery approaches are thoroughly reviewed elsewhere.^[^ ^66^, ^67^, ^68^, ^69^, ^70^, ^71^, ^72^ ^]^

If all barriers are successfully overcome, peptides enter the hepatic portal vein and transit to the liver, where they might undergo first‐pass metabolism, which can further reduce the amount of drug reaching the systemic circulation.^[^ ^6^ ^]^

Taken together, orally administered peptides must overcome a variety of challenging barriers to successfully reach systemic circulation. Even though the intestinal epithelium represents the major permeation hurdle and PEs can be leveraged to transiently alter this barrier, all barriers must be considered when formulating oral peptide therapeutics. This article focuses on overcoming the intestinal epithelium. Information on how to tackle digestion and mucus barrier can be found elsewhere.^[^ ^33^, ^34^, ^36^, ^38^, ^73^, ^74^, ^75^, ^76^, ^77^, ^78^, ^79^, ^80^ ^]^

## 3 Mechanisms of Permeation Enhancement

To improve the oral absorption of peptides, PEs must sufficiently but transiently perturb the intestinal epithelium barrier while exhibiting minimal local and systemic toxicity.^[^ ^50^ ^]^ Throughout the last decades, permeation enhancement has been explored by applying synthetic surfactants, bile salts, bacterial toxins, chelating agents, and medium‐chain fatty acids, which are thoroughly reviewed elsewhere.^[^ ^6^, ^15^, ^26^, ^28^, ^29^, ^81^, ^82^ ^]^ The next sections will focus on physiological fundamentals while highlighting possible new targets.

### 3.1 Paracellular Mode of Action

**Figure 2:**
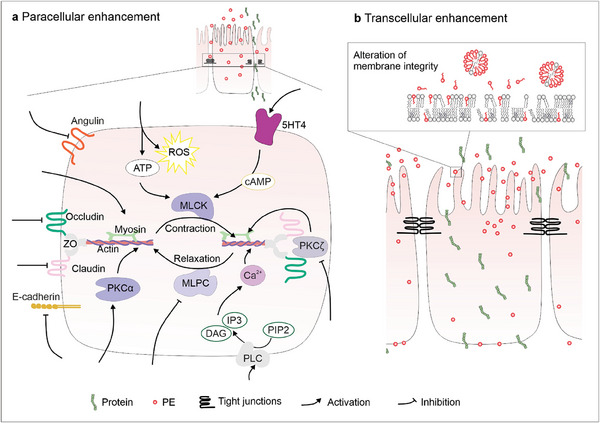
Different permeation enhancement mechanisms and possible targets. Schematic description of a) Paracellular permeation enhancement mechanisms. Arrow indicates that stimulation/activation and dead‐end blockage/inhibition lead to improved paracellular transport. Activation of myosin light chain kinase (MLCK) due to PEs generating reactive oxygen species (ROS), stimulation of the serotonin‐receptor 4 (5‐HT4), activation of protein kinase C (PKC)‐α, or phospholipase C (PLC), or myosin leads to cytoskeletal contraction. Inhibition of PKC‐ζ, angulin, occludin, claudin, or E‐cadherin induces cytoskeletal contraction; inhibition of myosin light chain phosphatase (MLCP) prevents cytoskeletal relaxation. b) Transcellular permeation enhancement by alteration of the membrane integrity.

Paracellular PEs increase permeability either indirectly by interference with mechanisms regulating TJs and/or AJs, or by alteration of TJ‐associated proteins (**Figure** **2**a).^[^ ^83^ ^]^

Specific alterations of the cytoskeleton can result in a temporary opening of TJs. These can be achieved either by binding to cofilin, an actin‐binding protein that regulates filament dynamics, and/or binding to actin filaments directly or by inducing the adenosine A1 receptor (A1R)‐mediated phosphorylation of actin‐binding proteins and MLC.^[^ ^84^, ^85^, ^86^ ^]^ As TJs also react to high intracellular amounts of toxic compounds such as ROS, intentionally releasing ATP and inducing ROS generation can be used to induce TJ opening.^[^ ^87^ ^]^ Other intracellular targets are enzymes that are involved in TJ and AJ modulation. One of those is PLC, which is located at the intracellular side of the cell membrane. It converts phosphatidylinositol‐(4,5)‐bisphosphonate (PIP2) into inositol‐(1,4,5)‐triphosphate (IP3) and diacylglycerol (DAG). These two mediators induce Ca^2+^ mobilization and protein kinase activation, ultimately leading to the contraction of the cytoskeleton and the opening of TJs.^[^ ^88^ ^]^ PKCs, which modulate proteins involved in TJ formation such as ZO‐1, are other possible targets for specific paracellular PEs.^[^ ^88^ ^]^ Activation of PKC‐α leads to actin polymerization and cytoskeletal contraction, which transiently opens TJs.^[^ ^89^ ^]^ The inhibition of PKC‐α‘s counterpart PKC‐ζ facilitates disruption and redistribution von ZO‐1 and occludin.^[^ ^90^, ^91^ ^]^

The balance of MLCK and MLCP activation determines the level of contraction of the actomyosin ring. Phosphorylation of MLC by MLCK triggers a conformational shift in enterocyte myosin II structure, resulting in cytoskeletal contraction and subsequent increase in paracellular permeability. This phenomenon may also involve the participation of ZO proteins and/or cingulin in mediating the effects.^[^ ^58^ ^]^ On the other hand, dephosphorylation by MLCP induces relaxation. By selectively inhibiting MLCP, levels of MLC phosphorylation can be indirectly increased without MLCK activation.^[^ ^92^, ^93^ ^]^ Indirect MLCK activation can also be achieved by stimulation of 5‐HT4 located at the cell membrane.^[^ ^94^ ^]^ Another approach to indirectly activate MLCK is to disrupt the delicate intra‐ and extracellular Ca^2+^ balance. Through chelation of extracellular Ca^2+^, the homeostasis between intra‐ and extracellular Ca^2+^ is disturbed, and Ca^2+^ is released from intracellular storage. Elevation of intracellular Ca^2+^ levels triggers a cascade of events, including the activation of calmodulin. Calmodulin activates MLCK leading to the disbandment of TJs and AJs, ultimately increasing epithelial permeability.^[^ ^88^ ^]^

All those approaches are reversible and maintain alternative TJ protein expression pathways. Enzyme modulation enables precise control of TJ opening, while simultaneously bypassing vital cellular components and thereby reducing toxicity risks. However, compared to strategies that directly target external TJ and AJ proteins, in this approach, successful traversal over membrane barriers of epithelial cells is required.

The development of PEs directly targeting TJ and AJ proteins started with the observation that natural toxins such as zonula occludens toxin (Zot) from *Vibrio cholerae* and *Clostridium perfringens* enterotoxin (CPE) trigger foodborne illness through TJ opening.^[^ ^95^, ^96^ ^]^ To date, as new TJ and AJ proteins are discovered, our understanding of both TJ and AJ mechanisms deepens and reveals numerous potential targets. Binding to the extracellular loops of different claudin proteins (including claudin‐1, −2, −3, −4, and −7) alters the paracellular barrier and increases epithelial permeability.^[^ ^95^ ^]^ Similar observations have been made for binding to the PDZ domain of claudins.^[^ ^83^ ^]^ In contrast to extracellular loops, the PDZ domain presents an intracellular target, that might be more challenging to reach. The elucidation of the occludin structure together with findings that the closing mechanism of TJs is based on a homologous interaction with another occludin has provided the foundation for the rational design of PEs targeting occludin.^[^ ^97^ ^]^ Peptide sequences mimicking either the first or the second extracellular loop motif of occludin were found to disrupt homophilic interactions between adjacent epithelial cells, thereby enhancing intestinal permeability.^[^ ^98^ ^]^ Cadherin is not considered as TJ protein, but the inhibition or disruption of its intercellular link connection (repetitive extracellular domains (EC 1–5)) between E‐cadherin molecules interferes with TJs. Binding to EC‐1 can disrupt E‐cadherin assembly and increase epithelial permeability.^[^ ^83^ ^]^

The direct targeting of TJ and AJ proteins has great potential for the development of novel PEs. One advantage is that there is no necessity to cross the epithelial cell membrane to achieve permeation enhancement. Yet, PEs should be designed to efficiently cross the mucus barrier. TJ/AJ opening must be transient, and the onset of the effect should be rapid (< 30 min). One possibility to enhance efficacy could be the combination of different direct and indirect TJ/AJ modulators. Further elucidation of the structure and function of TJs/AJs, and a better understanding of cytoskeletal control mechanisms, aided by empirical evaluations, could accelerate the design of new chemical and/or biological entities that target molecular mechanisms. Current paracellular PEs achieved pore diameters of the maximal space (100 Å = ca. 10 nm) between epithelial cells. The resulting gap is too small to permit the entry of pathogens such as lipopolysaccharides (molecular weight, MW > 100 kDa) and bacteria (size > 1 µm).^[^ ^99^ ^]^

### 3.2 Transcellular Mode of Action

Transcellular PEs facilitate transport directly through the cells themselves (Figure 2b).^[^ ^16^ ^]^ To date, most of these PEs are soluble surfactants—amphiphilic, surface‐active agents that interact with the plasma membrane altering its packing density and fluidity. By an action that is comparable to detergents, they are inserted into the lipid bilayer. This integration increases membrane fluidity, causes its expansion, and potentially leads to a localized disturbance of membrane integrity.^[^ ^100^ ^]^ Surfactants capable of enhancing drug permeation include medium‐chain fatty acids (MCFA), acylated amino acids, non‐ionic surfactants, bile salts, and acylcarnitines.^[^ ^101^ ^]^ Soluble salts of MCFA such as C8, C10, and sodium laurate (C12) are the most widely studied surfactant PEs.^[^ ^15^ ^]^ For most surfactants, the reversibility of permeation enhancement effects is time‐ and concentration‐dependent.^[^ ^102^, ^103^ ^]^

While there is no obvious correlation between surfactant structure and permeation enhancement efficacy, there are two inter‐dependent physicochemical parameters, the critical micelle concentration (CMC), and the hydrophilic‐lipophilic balance (HLB) that can be used to predict efficacy. Generally, soluble surfactants can exist in two forms: the free monomolecular form or micelles. The CMC marks the threshold above which micelles form, it is the highest concentration where monomers exist freely and varies with the buffer's composition. While the free form can directly interact with the cell membrane, micelles act as reservoirs that release free surfactant molecules to interact with the cell membrane. Due to the amphiphilic nature of surfactants, they can also interact with bile salts and phospholipids in the GI fluids and be integrated into existing bile salt/phospholipid micelles.^[^ ^104^ ^]^ For C10 it has been shown that variation in micelle composition affected the release of monomers from the micelles and insertion rates of C10 monomers into the cell membrane. Generally, the availability of free PE monomers to be incorporated into the membrane, which is important for permeation enhancement, depends on intestinal fluid composition and differences of the latter contribute to interindividual variability in the efficacy of PEs.^[^ ^105^ ^]^ The HLB value is a measure that quantifies the degree to which a surfactant is hydrophilic or lipophilic and serves as a guiding metric to estimate membrane insertion efficacy. Highly effective surfactants typically possess high CMC values ensuring that sufficient concentration of free surfactant is available for membrane interaction.^[^ ^106^ ^]^ Unfortunately, currently, CMC and HLB values for most commonly used PEs are not available. Comprehensive data may allow to predict the enhancement action based on HLB/CMC ratios, as it is crucial to find a balance where the CMC is paired with a sufficiently low HLB to ensure effective cell membrane interaction.^[^ ^107^ ^]^ Various CMC/HLB combinations can achieve comparable enhancements. For most ionic and non‐ionic surfactants, medium hydrophobic chain lengths (C8 to C12) show a favorable balance between solubility (high CMCs) and membrane penetration (moderate HLB), e.g. C10 that exhibits a relatively high CMC of ca. 25 mm and a moderate HLB of ca. 19.4. In contrast, surfactants with short chain lengths (C4 to C6) exhibit high CMCs but inefficient membrane insertion (too high HLB). Longer chains (C14 to C18) demonstrate greater membrane penetration efficiency (low HLB) but suffer from lower solubility (too low CMCs). Overall, despite the lack of reliable information on HLB and CMC values, most studies report significant enhancement action for surfactant PEs with medium hydrophobic chain lengths (C8 to C12) compared to longer or shorter chain lengths, irrespective of the hydrophilic head group.^[^ ^108^, ^109^ ^]^

Yet, it is noteworthy to state that most transcellular PEs were not rationally designed as surfactants with a high potential for membrane insertion. Most transcellular PEs were already well known, used as excipients or food additives, and then empirically found to have permeation enhancement effects. Besides altering the membrane permeability, most were additionally found to activate plasma membrane receptors and intracellular enzymes (PKC, MLCK), modulate intracellular mediators (Ca^2+^, calmodulin, and ATP), and/or selectively disrupt TJs.^[^ ^100^, ^108^, ^110^, ^111^ ^]^ The exact mechanism of action often remains partially understood, making the rational development of more effective substances highly challenging.

## 4 SNAC, C10, and C8 ‐ Oldies but Goldies?

To this point, > 50 clinical trials have shown that PEs, mostly surfactants, can increase the oral absorption of poorly permeable drugs, which is thoroughly discussed in several recent reviews.^[^ ^6^, ^15^, ^16^, ^81^, ^82^, ^109^, ^112^ ^]^ Notably, C8, C10, and SNAC stand out. They are among the most advanced intestinal PEs in terms of testing and characterization. They have been incorporated into oral formulations for over 20 years and have undergone more human trials than any other PE.^[^ ^103^, ^113^, ^114^ ^]^ Their well‐established safety in humans, C10 as a common food additive and, for SNAC, the FDA generally recognized as safe substance (GRAS) status gained in the approval of Eligen B12, renders them even more attractive. As C8 and C10 exhibit similar efficacy and mechanisms but C10 is more studied, the following section is focused on C10. C10 and SNAC are considered the gold‐standard for PEs, serving as the benchmark for newly emerging PEs.

### 4.1 C10

C10 is the sodium salt of capric acid and functions as a pH‐sensitive anionic surfactant acid. In gastric fluids with a pH significantly below its *pK*_a_≈5, it is inactive and exists as non‐ionized capric acid, which can reduce surface tension but lacks detergent action. However, in the small intestine, where the pH exceeds its *pK*_a_, C10 ionizes and acts as a potent detergent with high CMC.^[^ ^114^, ^115^ ^]^ Its mechanism of action is multimodal, enhancing transcellular and paracellular permeabilities. C10 has been the subject of many mechanistic studies, which are thoroughly reviewed elsewhere.^[^ ^103^, ^113^, ^115^, ^116^ ^]^ In brief, concentrations that cause significant changes in permeability coefficients of macromolecules (including peptides) and increase their oral BA are linked to mild mucosal damage and other signs of transcellular mode of action.^[^ ^116^ ^]^ In vitro studies additionally suggest the involvement of a paracellular mechanism.^[^ ^103^ ^]^ It is hypothesized that C10 activates PLC.^[^ ^116^ ^]^ However, even at the lowest concentrations needed to increase the permeability of macromolecules, plasma and mitochondrial membrane integrity is altered, indicating the involvement of the transcellular pathway.^[^ ^116^ ^]^ To date, the exact mechanism and the interplay between paracellular and transcellular mode ‐ whether it is an independent or consecutive process ‐ still remains unclear.^[^ ^117^ ^]^

Regardless of the exact pathways, there is a correlation between permeation enhancement and mucosal perturbation. Increased concentrations of C10 led to enhanced and prolonged absorption but also caused more damage to the epithelium. High concentrations of C10 used in oral formulations (≈500 mg) cause some degree of mild and reversible perturbations. However, their severity was shown to be similar to those caused by aspirin, alcohol, and spicy foods.^[^ ^118^, ^119^ ^]^ Additionally, studies in rats, dogs, and pigs have also demonstrated that exposure of the GI mucosa to C10 elicited no persistent morphological alterations. For example, the rat colonic epithelium exposed to 100 mm C10 following intestinal instillation was repaired within 30–60 min. The permeation enhancement effect of C10 was partially related to superficial epithelial damage caused in the first few minutes of exposure.^[^ ^120^, ^121^ ^]^ Interestingly, a study in rats performed at different pH values revealed that at the highest C10 levels tested (100 and 300 mm), the permeation‐enhancing effect of C10 was independent of its colloidal structure. However, at 50 mm, the efficacy was lower with micelles compared to vesicles. Overall, to achieve optimal macromolecule absorption enhancement high initial C10 concentrations (≥ 100 mm) are required, rendering local C10 concentration and total dose as critical factors.^[^ ^121^ ^]^

C10 is the main component of GIPET, an oral solid‐dosage form technology that was tested in over a dozen clinical studies.^[^ ^115^ ^]^ Importantly, various clinical trials revealed that the absorption‐promoting effects were transient, completed in under one hour, and no toxicity was detected in humans, even after subjects received multiple doses of GIPET.^[^ ^122^, ^123^, ^124^ ^]^ This technology could achieve a relative BA versus s.c. injection of 3.9–7.6% for low‐molecular‐weight heparin (LMWH; 4000–5000 Da). With desmopressin acetate (1069 Da), the BA reached 2.4% compared to 0.17% of the commercially available tablets without PEs (Minirin). Several Phase 1 studies were performed with fast‐acting insulin (IN‐105) and an oral antisense oligonucleotide (ISIS 104838). For the latter, an average oral BA of 9.5% relative to s.c. administration was achieved, however, the values ranged from 2–28%.^[^ ^125^ ^]^ A Phase 2 trial with once‐daily long‐acting basal insulin (I338) revealed a relative oral BA of 1.5–2% compared to the s.c. administered insulin glargine (Lantus, Sanofi). Even though the clinical performance regarding plasma glucose level reduction of the oral formulation was equivalent to s.c. insulin, the development was not continued.^[^ ^126^ ^]^ GIPET, along with further attempts to create an oral insulin was abandoned under the pretext of not being commercially viable. After > 40 years of attempts to develop an oral insulin formulation, most formulations suffered from large variability in oral BA. This together with the small therapeutic window of insulin makes it difficult to ensure a proper control of glycemia while avoiding side effects.

Recently, a Phase 2 clinical trial of an oral anti‐hypercholesterolemia treatment leveraging C10 as PE met its primary endpoint (change in low‐density lipoprotein (LDL)‐cholesterol between baseline and week 8).^[^ ^127^ ^]^ MK‐0616 is an oral PCSK9 inhibitor developed by Merck to lower LDL plasma concentrations. It is a macrocyclic peptide (1616 Da) with high potency and selectivity for PCSK9. In the Phase 1 clinical trial, 14 daily doses of either 10 or 20 mg MK‐0616 formulated with 360 mg C10 and 10 mg MK‐0616 with 180 mg C10 were administered. The trough MK‐0616 plasma concentration of 10 mg MK‐0616 formulated with 180 mg C10 and of the formulation with 360 mg C10 were comparable. An oral BA of around 2% was achieved. All three formulations (20 mg MK‐0616 + 360 mg C10, 10 mg MK‐0616 + 360 mg C10, and 10 mg MK‐0616 + 180 mg C10) exhibited similar % reduction from baseline LDL cholesterol.^[^ ^128^ ^]^

Overall, C10 has been shown to significantly increase the BA of macromolecules but with large variability. This, together with only moderate increases in oral BA represents the most important challenge, especially for peptide drugs which are not highly potent and exhibit a small therapeutic window.

### 4.2 SNAC

Historically, SNAC's development goes back to the 1990s, when it was developed as the main component of the Eligen carrier technology by Emisphere Technologies. Upon unsuccessful attempts to develop oral peptide formulations, SNAC was first approved in 2012 under medical food regulations in the oral vitamin B12 formulation Eligen B12.^[^ ^115^ ^]^ Subsequently, Novo Nordisk acquired Emisphere Technologies, obtaining ownership of Eligen and evaluating its compatibility with their insulin analogs and GLP‐1 agonists. Following the cessation of trials involving oral insulin, they focused on creating an oral version of the GLP‐1 receptor agonist, semaglutide.

Although SNAC, the synthetic N‐acylated amino acid derivative of salicylic acid (*pK*_a_≈5), shares structural features of MCFA, it does not display classical detergent‐like action in concentrations below its CMC (≈36 mm in Kreb's‐Henseleit buffer).^[^ ^115^ ^]^ While the mechanism of action has been extensively studied, it is not fully understood. Limitations and discrepancies of those studies and different hypotheses about SNAC's mode of action are explored in depth in other articles.^[^ ^15^, ^16^, ^103^, ^113^, ^114^, ^115^, ^129^ ^]^ SNAC was initially believed to enhance passive transcellular permeation by increasing lipophilicity of peptides via non‐covalent binding to peptides. However, to date, there is only limited and inconsistent data substantiating the initial chaperone hypothesis. Concurrently, there is poor evidence for a paracellular mechanism, leading to its classification as a transcellular PE.^[^ ^115^ ^]^ In various preclinical and clinical studies, high concentrations of SNAC (>> 40 mm in vitro and ≈300 mg in oral formulations) were needed to increase the permeability of drugs and/or drug surrogates, which is hypothesized to be based on membrane perturbation, fluidization, and non‐specific epithelial damage.^[^ ^103^, ^113^, ^130^ ^]^

Interestingly, Novo Nordisk proposed a different mechanism for the Rybelsus tablets, which contain 300 mg SNAC irrespective of the semaglutide dose.^[^ ^1^ ^]^ Rybelsus is formulated to target stomach absorption, achieved by designing the tablet with controlled erosion that remains in the lower stomach region. This formulation design aims at minimizing the dilution of both SNAC and semaglutide. SNAC can elevate the pH locally around the tablet in the stomach, protecting the peptide against pepsin. It can also enhance the solubility of the drug and promote monomerization in the nearby environment.^[^ ^131^ ^]^ The mode of transit of semaglutide through the gastric epithelium has been found to be predominantly transcellular. However, this mechanism might differ when using SNAC for the delivery of other drugs and/or targeting the small intestine. Until now, the exact mechanism of how SNAC enhances the permeability of peptides over the small intestinal epithelium remains unclear.^[^ ^103^, ^132^ ^]^

Despite its commercial success, the achieved oral BA of the Rybelsus formulation is rather low (0.4–1.2%) when compared to 87% BA of the s.c. injectable Ozempic. After singular‐dose administration, a significant number of patients had no measurable plasma drug concentrations. In the multiple‐dose trial, the intra‐individual day‐to‐day variability in semaglutide exposure was only 20–35% at steady state, while simultaneously an inter‐individual variability in semaglutide exposure of 65–85% was detected. This could stem from the low oral BA combined with a notable inter‐individual variability in the oral absorption of semaglutide. Yet, therapeutic plasma levels of semaglutide are reached at a steady state in almost all patients (96–98%) treated with once‐daily Rybelsus. Notably, this is due to the fact that the half‐life of oral semaglutide is 7 days, which leads to an overlap of consecutive once‐daily doses.^[^ ^133^ ^]^

Three decades of research assessing the safety and efficacy of C10/C8 and SNAC as PEs in preclinical and clinical settings reveal that they are comparable. All have been shown to increase oral BA of a range of peptide drugs to mean values of around 1% with high variability. Whether this increase in oral BA is sufficiently high to develop a commercial product mainly depends on the peptide characteristics. In the case of Rybelsus, it is semaglutide's extremely long half‐life and high therapeutic index that surpasses the limitation of low and variable oral BA. Unfortunately, most peptide drugs exhibit less favorable pharmacodynamics and pharmacokinetic properties and thus require more effective PEs to achieve higher (>5%) and more consistent oral BAs.

## 5 The Ideal Drug Candidate

Unfortunately, well‐established criteria like Lipinski's rule of five, which are reliable in predicting the feasibility of oral delivery cannot be applied to more complex drugs such as peptides.^[^ ^134^ ^]^ Lipinski`s rule of five considers factors such as MW, lipophilicity, hydrogen bond donors, and hydrogen bond acceptors. The rule suggests that compounds meeting certain criteria are more likely to be absorbed effectively when taken orally, resulting in adequate oral BA. However, peptides are fundamentally different from small molecule drugs regarding size and MW, structure, hydrophilicity, and degradation, placing them outside the boundaries of this rule.^[^ ^135^ ^]^ Lipinski's rule is primarily designed for molecules with MW < 500 Da, whereas many peptides have MW well above this threshold. Peptides often contain numerous polar and hydrophilic functional groups, including multiple amino acid residues with polar side chains. In contrast, Lipinski's rule emphasizes the importance of lipophilicity for oral absorption, the inherent high hydrophilicity of many peptide drugs can hinder permeation over the epithelial barrier. Overall, peptides face unique challenges when it comes to oral delivery, and predicting their oral BA requires considering factors specific to peptide chemistry, such as strategies to protect them from enzymatic degradation and enhance their transport across the intestinal barrier. For example, cyclic peptides are commonly associated with improved membrane permeability and metabolic stability, making them amenable to oral administration, in contrast to linear ones.^[^ ^136^, ^137^, ^138^ ^]^ For example, the cyclic peptide cyclosporin A is known to change its conformation depending on its solvent environment. This conformational flexibility seems to be key for the unusually high membrane permeability of cyclosporin A and other membrane‐permeable cyclic peptides. Indeed, conformationally constrained variants of those peptides have shown limited movement into, through, and finally out of the cellular membrane.^[^ ^139^ ^]^ On the other hand, there are examples of orally available linear peptides, that are either already on the market or in clinical trials, proving that the cyclic structure is no prerequisite. However, it is noteworthy to state that most of these linear peptides are rather small (≤ 1000 Da), and/or peptidomimetics. For larger and more complex ones (> 1000 Da and < 5000 Da) co‐formulation with PEs is needed to achieve therapeutic plasma levels.^[^ ^25^ ^]^

Even if conventional criteria cannot be applied directly, there are certain peptide characteristics that favor the successful development of oral formulations, especially when formulated with suitable PEs. In the following section, we outline the key features that peptides should possess to be suitable for the oral route (**Table** **2**).

**Table 2:** Criteria for selecting peptides suitable for oral delivery.

– Injection‐based therapy requires frequent and/or inconvenient dosing regimens.
– Injection‐based therapy elicits pain or discomfort and/or requires administration by a healthcare professional.
– Cost of goods sold (COGs) of oral formulation lower/comparable to injectables.
– MW lower than 5 kDa.
– High potency (low dose required;) and long half‐life.
– High therapeutic index.

Most (> 96%) peptide drugs that are either already approved or in clinical trials are developed for parenteral administration.^[^ ^29^, ^81^ ^]^ To be clinically considered for oral reformulation, current injection‐based therapies should require frequent and/or inconvenient dosing regimens, and/or elicit pain or discomfort. The final oral formulation must lead to similar therapeutic efficacy compared to the injectable counterpart. Other factors that impact the feasibility of oral delivery are pharmacodynamic and pharmacokinetic properties. The therapeutic index, potency, plasma half‐life, and oral BA of the peptide drug are important parameters because oral delivery will generally result in more variable and lower blood concentration than parenteral delivery. If the half‐life is too short and/or potency too low, and oral BA is not high enough, oral delivery may lead to erratic therapeutic responses. The high variability encountered with current formulations also implies that the drug should possess a large therapeutic index to avoid potentially dangerous side effects resulting from overexposure.^[^ ^81^, ^140^, ^141^ ^]^

Third, the structure and physicochemical properties of the peptide itself influence the degree of degradation, mucus permeation, and absorption. Peptides intended for oral delivery should ideally exhibit some resistance to the digestion process.^[^ ^142^ ^]^ Enzymatic stability strongly depends on the sequence and structure of the peptide. Even though various in vitro and ex vivo studies were performed to assess the stability and enzymatic degradation of peptide‐based drugs, there is a lack of data on recently approved peptide‐based drugs. Yet, there are some generally accepted benchmark values.^[^ ^27^, ^77^ ^]^ Small peptides (< 3 kDa), including those with cyclic structures, are more stable.^[^ ^25^, ^76^, ^79^ ^]^ Proteolytic cleavage sites within the peptide can be identified through stability investigations and the analysis of metabolites. Stability against proteolytic degradation is generally enhanced by backbone modification of the peptide, such as replacing L‐amino acids with D‐amino acids, introducing methyl‐amino acids, and/or integrating β‐amino acids and peptoids (synthetic oligomers/polymers similar to peptides but with their side chains attached to the nitrogen atom of the backbone, rather than the alpha carbon). Furthermore, the insertion of D‐amino acids can also increase the plasma half‐life of the peptide once absorbed. Serum degradation and elimination from the systemic circulation can also be reduced through polymer grafting (e.g., poly(ethylene glycol), PEG). As mentioned above, another very common approach to improve proteolytic stability as well as cell‐permeability is the cyclization of peptides. Strategies to achieve this include head‐to‐tail, backbone‐to‐side chain, and side chain‐to‐side chain cyclization.^[^ ^143^, ^144^ ^]^ Acid‐base properties, hydrophobicity, and terminal amino acid composition also influence GI tract stability. The terminal amino acid composition affects the resistance to hydrolysis. Peptides with the C‐terminal amino acid lysine or arginine are more likely to be cleaved by trypsin while peptides with C‐terminal proline, glycine, alanine, or serine are generally more resistant.^[^ ^3^, ^29^, ^79^, ^81^, ^145^ ^]^ At this point, it is noteworthy to mention that the cyclic peptides cyclosporin A, volclosporin, desmopressin, and octreotide possess sufficient stability in the gastric and intestinal environment.^[^ ^79^ ^]^ This stability is advantageous and fosters the development of their oral formulations.^[^ ^27^, ^141^ ^]^ An example of rational protein optimization is semaglutide, which was designed to exhibit enhanced stability in the plasma compared to native GLP‐1 and other earlier GLP‐1 analogs. It has structural modifications that confer resistance to enzymatic degradation by dipeptidyl peptidase‐4 and neutral endopeptidases, enzymes that typically degrade GLP‐1 peptides. The attachment of a fatty acid chain plays a role in its interaction with the GLP‐1 receptor and enables its binding to albumin, mainly prolonging the half‐life by reducing renal clearance but also contributing to GI stability by protecting it from enzymatic degradation in the GI tract.^[^ ^1^, ^131^ ^]^

Peptides intended for oral delivery with a MW below 5 kDa are preferred candidates for formulation with both transcellular and paracellular PEs due to their potential for sufficient absorption through the intestinal epithelium.^[^ ^4^ ^]^ Absorption is enhanced if these peptides possess some degree of lipophilicity. The integration of lipophilic or amphiphilic amino acids, such as leucine, phenylalanine, or tryptophan, and chemical modifications like fatty acid conjugation, can enhance lipophilicity. Most larger peptides are restricted from using the paracellular pathway and require alternative mechanisms, such as transporter‐mediated uptake, to cross the intestinal barrier.^[^ ^25^, ^146^, ^147^, ^148^ ^]^

When considering interaction with mucus, there are two favorable scenarios. The first relies on efficient diffusion through the mucus layer.^[^ ^149^ ^]^ Peptides with smaller MW and a compact structure generally diffuse better through the mucus layer. Proteins with minimal or weak interactions with mucin fibers can penetrate the mucus layer more effectively. Minimizing the binding affinity of proteins to negatively charged MUCs, without causing repulsion, can reduce their entanglement within the mucus mesh and enhance diffusion.^[^ ^33^, ^34^, ^150^, ^151^ ^]^

While seeming contradictory, the second strategy relies upon increasing the mucoadhesion of the peptide drug. Mild mucoadhesive properties can aid in mucus diffusion. By interacting with the mucus layer, mucoadhesive peptides can avoid rapid clearance and reach proximity to the underlying epithelium, enhancing the absorption potential. This can be achieved by either introducing positively charged or thiolated amino acids, such as arginine or cysteine, or by modifications that promote hydrogen bonding with mucin glycoproteins.^[^ ^38^, ^73^, ^78^ ^]^

The ideal peptide drug candidate for oral delivery is therefore a molecule with relatively low MW that is highly potent, stable in the GI tract, and exhibits a long half‐life. Such a peptide might even reach therapeutic levels without the need for a PE. An example is desmopressin acetate, which is formulated as a conventional tablet devoid of PE. While its half‐life is quite short (ca. 3 h), it is small (≈1 kDa), extremely potent, relatively stable in the GI tract, and endowed with a large therapeutic window (LD_50_ (i.v.) in mice = 2 mg kg^−1^). Nevertheless, its oral BA remains quite low (0.08–0.17%).^[^ ^152^ ^]^ This is sufficient in this case but for other peptides that are commonly 50‐ to 200‐fold less potent, there is the necessity of improving permeation.^[^ ^29^ ^]^

## 6 What is Coming Next?

Given the abundance of studies related to PEs, one might ask what is coming next and which new strategies may overcome current limitations.

Over the last few years, there has not been a lot of research investigating new PEs. Of those, only a few were tested in vivo with still fewer being assessed in humans or large animal models. Assessing the performance of PEs in cell culture models or static ex vivo models has limited relevance as these models do not account for the actual dynamic environment in the GI tract. Dilution of the drug and excipient in intestinal fluids and the necessity of temporal synchronization of the drug and the PE are not considered. Although the results can often not be used to predict in vivo efficacy, in vitro testing using monolayers of human colon carcinoma (Caco‐2) cells remains the center of PE research and a valuable tool to first screen PE effectiveness. Permeability of drugs or drug‐surrogates (fluorescently labeled macromolecules with defined MW) and PE efficacy as a function of reduction of transepithelial resistance (TEER) can be used to compare novel PEs with C10 or SNAC.^[^ ^153^ ^]^ Caco‐2 monolayers are, if carefully controlled, highly reproducible in vitro models.^[^ ^154^ ^]^ Usually, %TEER decrease compared to control values is recorded as a function of time, and/or the apparent permeability coefficient (P_app_) of a specific drug or drug surrogate is assessed. P_app_ is used to calculate the enhancement ratio, which is the ratio of the P_app_ achieved with PE treatment to the P_app_ of the control group. Beside cell studies, animal models, primarily rodents, are commonly employed to determine the performance of PEs regarding pharmacokinetic and pharmacodynamic parameters. However, evaluating and optimizing oral formulations in rodents has limited benefits as those models do not represent the human physiology of the GI tract. Results on peptide BA obtained with mice or rats often cannot be transferred directly to humans. Rats and mice have a less acidic stomach pH, and faster gastric emptying and intestinal transit times compared to humans. Their gut microbiota composition and enzymatic activity also differ. Additionally, they have a relatively (body weight adjusted) larger surface area available for adsorption in the small intestine and bile production and secretion are different.^[^ ^155^ ^]^ All of these influence the dissolution, stability, and absorption rate of the peptide, leading to variable study outcomes and most likely to an overestimation of oral BA. Consequently, one major challenge in the development of new PEs is the lack of reliable, robust, and physiologically relevant in vitro and ex vivo models. Currently, it is not clear how to efficiently discover efficient PEs, and improvements in screening methodologies are critically needed. Commonly used methods often fail to accurately predict the efficacy of PEs in enhancing oral BA of macromolecules because they do not adequately replicate the complex processes and barriers present in vivo. This gap in methodology was recently highlighted by Emeh et. al.^[^ ^156^ ^]^ Improving the predictability might be achieved by using large porcine GI tissue explants in a high throughput screening campaign. This approach led to the identification of formulations that were proven effective in pigs.^[^ ^157^ ^]^ Yet, the scarcity of human and even large animal data on PEs, and the common practice of using rodents, sometimes with the formulation directly instilled in the large intestine (which is justified when studying mechanistic aspects), complicate the accurate assessment of PEs under conditions that mimic human GI tract dynamics during oral drug intake. Historically, it has been shown that assessing oral formulations as quickly as possible in humans or at least in large animal models such as pigs and dogs is essential for success.^[^ ^16^ ^]^

Until now, PEs with little to moderate permeation enhancement effects have been preferred over highly effective ones likely due to safety concerns. These include irreversible epithelial cell damage and/or irreversible or long‐lasting tight junction opening (**Table** **3**). Other commonly raised issues are the risks of promoting systemic exposure to microorganisms, toxins, and pathogens, to trigger immune reactions, and cause sepsis or inflammation. However, to date, there is no evidence of pathogen, lipopolysaccharides, and/or exo‐ and endotoxins co‐absorption, at least to clinically relevant levels.^[^ ^158^ ^]^ This is further underlined by the fact that for various PEs that have been tested in clinical trials, including high amounts of C10 (550 mg, once daily for 8 weeks), which is one of the most effective PE, no significant adverse effects besides sporadic mild to moderate GI side effects have been observed.^[^ ^126^, ^158^ ^]^ Similar clinical side effects have been reported for Rybelsus. While the cause of GI side effects such as nausea, vomiting, diarrhea, abdominal pain, and loss of appetite can stem from either the drug itself or SNAC, they are not giving rise to concerns about SNAC as PE. Yet, they are unpleasant for the patient and might impact the clinical effects of Rybelsus on weight loss.^[^ ^159^, ^160^ ^]^

**Table 3:** Safety concerns versus clinical safety profile.

Common safety concerns	In vitro evidence	Reference	Ex vivo/in vivo evidence	Reference
Intestinal cell toxicity	Cytotoxicity (Caco‐2); C10, Labrasol	[115, 164]	None, no histological or morphological alterations of intestinal tissue	[118, 165]
Permanent/sustained TJ opening and/or membrane alteration	Sustained TJ opening; Pelargonidin/membrane alterations; C10	[103, 161, 166]	None, transient and reversible (<1 h) barrier alterations	[124, 166]
Co‐absorption of pathogens	–	–	None, no immunologic or inflammatory side effects in clinical trails	[122, 123, 124, 126, 159, 160, 167]
Immune reactions and/or inflammation				

The generally favorable toxicity profiles of common PEs might be due to short residence time in the GI tract, dilution effects, and highly efficient repair mechanisms of the small intestine in vivo. In humans, permeation enhancement effects of PEs such as C10 have been shown to be transient and do not last longer than one hour. No toxicity was detected in humans, even after subjects received multiple doses of PE‐containing formulations.^[^ ^122^, ^123^, ^124^ ^]^ More so, most safety concerns are based on in vitro or ex vivo results such as detected toxicity of Caco‐2 cells, irreversible reduction of TEER, prolonged or irreversible TJ opening, and morphological changes of the exposed intestinal tissue.^[^ ^111^, ^161^, ^162^, ^163^ ^]^ Yet, none of those are directly translatable to in vivo conditions. Overall, it seems that even though reversibility of the permeation effect, independently if membrane perturbation or tight junction opening, is mandatory to ensure safety, it is almost impossible to predict the extent of the effect and safety in vivo from in vitro and ex vivo experiments. In most cases even if pre‐clinical experiments revealed potential risks, none of those were observed in large animal models or humans.

However, it remains unclear whether the efficacy of intestinal epithelial damage‐repair is sustained during chronic therapy, especially when more effective PEs are used. Ideally, novel PEs would be as safe but more effective than C10 and SNAC, transiently open tight junction or perturb membranes within seconds to minutes after release for a maximum of one hour, with little to no absorption into the systemic circulation.

Another challenge is the low and highly variable oral BAs, especially for peptides with poor GI tract stability and permeability, narrow therapeutic indices, and short half‐life. One approach to tackle these issues is the use of advanced drug delivery systems. Various approaches have been investigated over the last few years.^[^ ^14^, ^112^, ^168^, ^169^, ^170^ ^]^ These include gastroretentive systems enhancing the residence time in the stomach, multiparticulate systems like microparticles and nanoparticles aiming for peptide protection and controlled release, and microneedle‐based systems designed to overcome the epithelial barrier.^[^ ^10^, ^11^, ^12^, ^13^, ^171^ ^]^ Although microneedle‐based devices have shown encouraging results in both in vitro and in vivo studies, their complexity, moderate reliability, and associated production costs, as well as their uncertain long‐term effects raised concerns about their practicality for clinical use.

One prominent challenge arises from the complexities and costs associated with developing new excipients. If researchers manage to create novel PEs, there exists a substantial burden associated with demonstrating their long‐term safety, efficacy, and mechanistic actions before regulatory approval can be obtained, demanding extensive preclinical and clinical testing. This represents a major barrier to driving development, especially for novel PEs with comparable or only moderately improved efficacy versus well‐established ones. As a consequence, the pharmaceutical industry often opts for using previously approved PEs with established records of safety and efficacy as those offer a more predictable and streamlined path to regulatory approval.^[^ ^82^, ^124^, ^172^ ^]^ In such cases, intellectual property and patent protection are achieved for the formulation of drugs and PE as exemplified by Rybelsus.^[^ ^1^ ^]^ This reliance on known PEs, however, may limit the development of new PEs that could potentially yield more efficient therapies. From a regulatory perspective, programs such as the FDA's PRIME initiative, which proposes a pathway for approving excipients independently of their associated bioactive molecules, could foster the development of novel PEs.^[^ ^173^ ^]^

However, we believe that for truly groundbreaking PEs, allowing for oral BAs in the double‐digit range > 10%, there would be enough incentive in this field to bring a new system to the market. Overall, in our opinion the major issue for the development of new PEs is not presented by intellectual property or patent issues, nor by high development costs, but the lack of truly potent PEs that significantly outperform established ones.

### 6.1 Paracellular PEs

Inspiration for developing potentially better paracellular PEs can be drawn from toxins targeting TJ and AJ proteins in adjacent epithelial cells or leveraging rational drug design to develop new specific TJ modulators instead of screening families of excipients for unspecific permeation enhancement effects.

*Clostridium perfringens* iota‐toxin (Ib) targets angulin‐1, which is one of the two main proteins of tricellular TJs and was used as a blueprint to develop angubindin‐1 (Ib421‐664). In vitro, angubindin‐1 reduced the TEER to 50% after a 24 h treatment period and doubled permeability of fluorescein isothiocyanate–dextran with a MW≈4 kDa (FD4) and fluorescein isothiocyanate–dextran MW≈10 kDa (FD10) through Caco‐2 monolayers.^[^ ^174^ ^]^ The necessity of a long incubation time (24 h) renders this approach impractical. Unfortunately, this phenomenon has been also seen for other TJ modulators and represents one major limitation that needs to be overcome to successfully develop TJ‐specific PEs.

The peptide PN159 is a TJ modulator with a very fast onset of action that has been suggested to bind to claudin. One minute after treatment with 10 mm PN159, TEER of Caco‐2 cells dropped to 42% of the control value, reached 16% after 5 min, and 1.4% after 30 min of incubation. PN159 increased the permeability of fluorescein (MW ≈376 Da) by 200‐fold and albumin (MW ≈65 kDa) by 30‐fold.^[^ ^175^ ^]^ Compared to C10, 1000‐fold lower concentrations of PN159 were used to achieve even better results in terms of TEER reduction and permeation enhancement in vitro. Impressively, a complete recovery of the monolayer was shown after 24 h, which is not the case for C10. After an incubation period of 2 h, TEER values recovered within 24 h only if < 5 mm C10 was used, while there was no recovery at higher concentrations (> 8.5 mm).^[^ ^103^ ^]^ Even though the in vitro data suggested PN159 being worth further development, to the best of our knowledge, this molecule was not further tested as oral PE. While studies assessing its potential for oral delivery are from 2019, there is an abandoned patent and several publications investigating PN159 for intranasal peptide administration from 2006. These also include studies in rabbits, where the absolute intranasal BA of the peptide YY 3–36 was increased from 0.3% to 14% in the presence of 50 µM PN159.^[^ ^176^, ^177^ ^]^ Angubindin‐1 and PN159 were both developed using a phage display library.^[^ ^178^ ^]^ Leveraging biotechnological tools to identify new PEs that address newly identified targets, therefore presents a valuable tool for future studies.

In a Caco‐2 monolayer screen of 51 substances, 1‐phenylpiperazine (PPZ) was identified as an efficient PE with low cytotoxicity. PPZ modulates TJ complexes via interaction with 5‐HT4. Binding to the receptor induce the release of cAMP which in turn activates MLCK.^[^ ^94^ ^]^ After 20 min, 6 mm PPZ reduced the TEER to < 20% of control values and increased the permeability of FD4 6‐fold.^[^ ^94^ ^]^ A study performed by Fein et al. reported similar results. PPZ (6.5 mM) reduced TEER to ≈40% after 30 min and induced a nearly 10‐fold increase in FD4 permeability and a 2‐fold increase for FD10. In mice, 65 mg kg^−1^ (195 mm) PPZ delivered by intestinal injection, caused a 10‐fold increase in FD4 blood plasma levels, but changing the application route to oral gavage rendered PPZ ineffective. However, efficacy was restored when mice received an oral gavage of 10% sodium bicarbonate solution 15 min before the PPZ treatment to avoid protonation of PPZ (**Table** **4**).^[^ ^99^ ^]^ Despite its initial promise with similar efficacy as C10 in vitro, PPZ might not be worth further investigations. A pretreatment preceding the administration of the peptide would not be practical and preventing protonation of PPZ (*pK*_a_ = 8.94)^[^ ^179^ ^]^ by altering the intestinal pH might be more difficult to achieve in larger animal models or humans. Besides, activation of 5‐HT4 causes the alterations of intestinal epithelial ion transport functions and of the vital enteric 5‐HT system, bearing the potential of severe side effects.^[^ ^94^ ^]^

**Table 4:** Pharmacokinetic data.

PE	API	Model	Exposure	vs. C10	Reference
PPZ	FD4	Mouse, intraintestinal	10‐fold vs. PBS	–	[94, 99]
Pelargonidin	Insulin	Mouse, gavage	N.A.	?	[166]
Sucrose laurate	Insulin	Rat, intrajejunal	1.3–2.5% BA^a)^	≈	[164]
Labrasol	Insulin	Rat, intrajejunal	6.75% BA^a)^	≈	[165]
CAGE	Insulin	Rat, intrajejunal	51% BA^a)^	+	[189]

^a)^ Relative to s.c. potency compared to C10: – less potent, + more potent, ≈ similar, ? unknown.

Pelargonidin, an anthocyanidin derived from strawberries, was recently identified as a promising PE. The suggested mode of action is paracellular. 1 mg mL^−1^ (≈3.5 mm) pelargonidin reduced TEER to < 20% compared to control values after 1 h incubation and improved calcein (MW≈600 Da) permeability 50‐fold. In mice, pelargonidin moderately increased FD4 plasma levels when pelargonidin and FD4 were applied simultaneously compared to a PBS control (1.8 vs. 1.2 µg mL^−1^), but a more pronounced effect (2.75 µg mL^−1^) was achieved when the animals were exposed to the PE 1 h before administration of FD4. Treatment with enteric capsules containing insulin, the protease inhibitor aprotinin, and 40 mg kg^−1^ pelargonidin moderately but significantly decreased blood glucose levels 4 h after administration compared to oral insulin without PE. As a readout, blood glucose levels can be influenced by various external factors such as food intake, physical activity, and stress.^[^ ^166^ ^]^ Unfortunately, the oral BA was not reported in this work. To ultimately evaluate the potential of pelargonidin as intestinal PE, studies in large animal models or humans would be needed.

In recent years, very few efforts have been made to develop new paracellular PEs. Various old paracellular PEs, such as C‐terminal fragments of *Clostridium perfringens* enterotoxin (e.g., C‐CPET), derivatives of Zot (e.g., AT1002), occludin peptides (OCC_2,_ OP90‐113, OP90‐135, and OP90‐103), permeable inhibitors of phosphatase (PIP) 250 and 640, and viral protein 8 (VP8) are repetitively discussed ever since their discovery.^[^ ^15^, ^180^ ^]^ While routinely stated as highly promising approaches, not one proceeded to clinical testing. Reasons for this may include instability of peptide‐based PEs, failure in reaching intracellular or basolateral targets, and limited permeation enhancement efficacy. Additionally, only a few revealed efficient in vitro effects, and not one outperformed gold‐standard PEs in vivo.

### 6.2 Transcellular PEs

Similarly, only a few new transcellular PEs emerged in the last years. Of those, none exhibited superiority over C10 (Table 4). Sucrose laurate (SL), a food additive, was found to hamper membrane integrity leading to TJ opening. The concentrations that enhanced flux in Caco‐2 cells also caused cytotoxicity. 1 mm SL reduced TEER < 20% within 20 min incubation and increased ^14^C‐mannitol permeability by 10‐fold. In rat intrajejunal instillations, 50 and 100 mm SL co‐administered with insulin achieved a relative to s.c. BA of 1.3% and 2.5%, respectively. In the same experimental setup, 50 and 100 mm C10 achieved 4.4% and 3.3% relative to s.c. BA, respectively.^[^ ^164^ ^]^ The authors concluded that SL can therefore be added to the list of potential PEs but no further investigations have been reported since.

Another well‐known excipient that gained attention as a PE is Labrasol which constitutes a mixture of mono‐, di‐ and triglycerides and mono‐ and di‐fatty acid esters of PEG‐8 and free PEG‐8, with caprylic (C8)‐ and capric acid (C10) as the main fatty acids. Originally, Labrasol was investigated as a compound of self‐microemulsifying drug delivery systems (SMEDDS) to improve oral BA of lipophilic drugs. SMEDDS are isotropic mixtures of drugs with oil, surfactant, and co‐surfactant which can form oil‐in‐water (o/w) microemulsions. It was hypothesized that SMEDDS act by altering membrane permeability and open TJs. In vitro studies confirmed the redistribution of ZO‐1 and actin upon 2 h exposure to SMEEDDS containing Maisine 35‐1, Kolliphor EL, Labrasol, and Transcutol (diethylene glycol monoethyl ether).^[^ ^181^ ^]^ In another study, an aqueous solution containing 1% of Labrasol reduced TEER to 40% within 30 min and increased mannitol flux by 30‐fold.^[^ ^181^ ^]^ In rat intrajejunal instillations, 40 mg mL^−1^ Labrasol co‐administered with insulin achieved a relative to s.c. BA of 6.7%.^[^ ^165^ ^]^ Labrasol was also tested with MK‐0616 in a Phase 1 clinical trial (10 to 300 mg of MK‐0616 formulated with 1800 mg Labrasol). Despite C10 (200 mg MK‐0616 and 360 mg C10) and Labrasol (200 mg MK‐0616 and 1800 mg Labrasol) showing comparable permeation enhancement effects that increased overall drug exposure by 2‐ to 3‐fold, the development progressed with C10.^[^ ^127^, ^128^ ^]^

AMT‐101 and AMT‐126 developed by Applied Molecular Transport, operating as Cyclo Therapeutics, Inc. as of 21^st^ September 2023 (San Francisco, USA), are formulations leveraging toxin‐inspired, peptide‐based transcellular PEs. Both are based on a recombinant biologic fusion protein of human interleukin‐1 and interleukin‐22, respectively, and a toxin‐based carrier protein that mediates transcytosis through intestinal enterocytes. Despite oral IL‐10 (ATM‐101) successfully concluding a Phase 2 trial for the treatment of chronic pouchitis and AMT‐126 having completed a Phase 1 trial, the development seems to be discontinued.^[^ ^182^, ^183^ ^]^ The PE mechanism is based on a non‐toxic form of cholix. Cholix is an exotoxin secreted by *Vibrio cholerae* which can traverse the epithelium via vesicular transcytosis.^[^ ^182^, ^184^ ^]^ However, the aim of those formulations differs significantly from conventional oral delivery approaches. The overarching goal is to overcome the challenge of local administration of interleukins to the region below the intestinal epithelium (lamina propria) without significant systemic exposure. Therefore, the amount that is required to cross the intestinal epithelium and achieve local effects is lower than what would be needed to achieve therapeutic systemic effects. Applied Molecular Transport seems, however, to have ceased its drug delivery activities following the recent merger with another company.^[^ ^185^ ^]^

An approach aiming for systemic peptide delivery which is currently tested in clinical trials is the Peptelligence technology of Enteris Biopharma (Boonton, USA). It is based on a multimodal mode of action comprising an enteric coating, a sub‐coat that allows simultaneous excipient release, citric acid granules, and in some cases surfactant‐type PEs (an acylcarnitine or bile salt). Acylcarnitines and bile salts are amphiphilic, surfactant‐type transcellular PEs that alter the cell membrane and can also affect TJ proteins such as ZO‐1 and claudins‐1, ‐3, and ‐5.^[^ ^158^ ^]^ Already in 2013, it was tested in a clinical trial to deliver recombinant human parathyroid hormone [rhPTH(1‐31)NH2] orally but was inferior to the s.c. drug and was not further developed.^[^ ^167^ ^]^ Another attempt in 2015 with oral salmon calcitonin (formerly known as TBRIA) was successful and met clinical endpoints in a Phase 3 study (ORCAL trial).^[^ ^186^ ^]^ However, TBRIA was never approved possibly due to the lack of efficacy of the drug in preventing fractures,^[^ ^187^ ^]^ and a coincidental warning from the FDA regarding the use of nasal salmon calcitonin.^[^ ^188^ ^]^ To date, the Peptelligence technology is also part of clinical trials with leuprolide and difelikefalin. Enteris Biopharma is developing oral leuprolide (Ovarest) and Cara Therapeutics (Stamford, USA) oral difelikefalin (Korsuva).^[^ ^28^ ^]^

### 6.3 Ionic Liquids

Ionic liquids are salts comprising organic cations and anions with melting points below 100 °C. One of the most prominent examples is choline geranate (CAGE), which is generated by mixing choline bicarbonate and geranic acid (molar ratio of 1:2).^[^ ^190^ ^]^ CAGE was found to increase the paracellular permeability of insulin and to aid in overcoming the digestion and mucus barrier. An extremely high oral BA of insulin (51%, relative to s.c.) was reported. However, Insulin‐CAGE group (5 U kg^−1^ insulin) was compared to fewer amounts of insulin injected s.c. (2 U kg^−1^) and overall insulin doses used were extremely high. Additionally, the first time point in the PK profile of the s.c. insulin (2 U kg^−1^) group was rather late for s.c. application (1 h), which could have led to an overestimation of oral BA. Treatment of fasted rats (oral gavage followed by s.c. administration of metoclopramide to stimulate gastric emptying) with enteric capsules of insulin and CAGE (10 U kg^−1^ insulin‐CAGE) resulted in a blood glucose drop comparable to s.c. injected insulin (2 U kg^−1^ insulin).^[^ ^189^ ^]^ Unfortunately, the oral BA was not reported. To ultimately evaluate the potential of CAGE, thorough toxicity studies as well as studies in large animal models and humans including precise determination of pharmacodynamic and pharmacokinetic parameters are needed.

### 6.4 Micro‐ and Nanoparticles

Already back in the 1980s, nanoparticles (NP) were employed to enhance oral peptide delivery.^[^ ^191^, ^192^ ^]^ One of the first attempts was made using insulin NPs (= intermolecularly crosslinked insulin molecules produced by crosslinking with glutaraldehyde). Oral administration of 350–700 mg kg^−1^ of NPs to rats reduced blood glucose 3 h after administration to 15–20% of the initial value, while there were no effects for saline or free insulin.^[^ ^191^ ^]^ However, the required doses of NPs made the further development of a commercially successful product unfeasible.

Despite > 40 years of continuous attempts, no major success has been achieved despite numerous studies reporting enhanced oral BA through NP delivery in rodents.^[^ ^37^, ^67^, ^69^, ^193^ ^]^ NP permeability over the epithelium remains the major limitation.^[^ ^194^ ^]^ In our view, while fine‐tuning particle size and surface characteristics, including the integration of targeting ligands, may improve NP transport across cellular barriers, the oral BA of the peptide cargo will still be limited. One reason for this is that only low amounts of NPs can be efficiently transported over the epithelial barrier. This can additionally be exacerbated by inadequate peptide loading efficiency.^[^ ^67^, ^69^, ^70^, ^71^, ^193^ ^]^

While the hepatic‐directed vesicles (HDV) were initially developed for oral insulin delivery by Diasome (Cleveland, USA),^[^ ^28^, ^195^ ^]^ they stopped their efforts on oral insulin after a first Phase 2 study due to inconsistent dose responses.^[^ ^196^ ^]^ The liposomal vesicles with a diameter of 150 nm, contain a specific hepatocyte‐targeting molecule in their phospholipid bilayer. Diasome now states to have oral HDVs for incretin delivery in their preclinical pipeline.^[^ ^197^ ^]^ It will be very interesting to see what the outcome of future studies will be since liposomes are not very stable in the GI environment, especially in the presence of bile salts.^[^ ^198^ ^]^ It is also possible that in this case the lipids from the liposome formulation act as PEs.^[^ ^199^ ^]^

Despite using NPs as delivery systems facilitating the overcoming of GI barriers, recently, negatively charged inorganic silica NPs have been found to exhibit permeation enhancement potential. Silica NPs with a size of 50 nm showed similar permeation enhancement efficacy as C10 in vitro. For the in vivo experiments, mice were orally gavaged with 100 mg kg^−1^ 50 nm silica NPs 2 h before the oral gavage of drug surrogates or drugs. While increased BA for insulin and exenatide was observed, all in vivo experiments required the administration of high amounts of silica NPs 2 h before drug dosing.^[^ ^200^ ^]^ This limitation together with missing evidence in large animal models, questions the potency of silica NPs as PEs and its translatability into clinical practice.

## 7 Conclusion

The successful development of Rybelsus and Mycapssa are remarkable achievements, however, the peptides used exhibit the most favorable characteristics for the development of an oral formulation, and still, the BA remains relatively low. There is plenty of room for further enhancement. Most oral peptide delivery technologies that are currently in clinical trials still use established gastrointestinal PEs with moderate permeation enhancement efficacy that have a history of safe use in man.

There must be a shift to the rational design of drug‐like PEs with well‐known modes of action. Recently acquired biopharmaceutical insights together with the discoveries of novel targets should be leveraged to develop more advanced PEs. To evaluate novel PEs, more robust preclinical data including the systemic evaluation of BA, generated in large animal models, as well as more robust, physiologically relevant in vitro models are needed. PEs with different modes of action may be combined and used together to achieve synergism. Simultaneously, to fully mitigate safety concerns about highly effective PEs, thorough toxicological studies are needed. Next‐generation PEs should aim to achieve oral BA >> 5%, ideally, in the double‐digit range (> 10%). This would allow the development of oral formulations of a much wider selection of peptides and other drugs that sit outside of Lipinski's rule of five, ultimately ushering in a new era of oral peptide delivery.

## Conflict of Interest

J.C.L. is a shareholder of Obaris AG, a company developing transbuccal delivery systems.
